# Validity of Micro-Data Loggers to Determine Walking Activity of Turkeys and Effects on Turkey Gait

**DOI:** 10.3389/fvets.2018.00319

**Published:** 2019-01-31

**Authors:** Rachel Stevenson, Hillary A. Dalton, Marisa Erasmus

**Affiliations:** ^1^Department of Animal Sciences, Purdue University, West Lafayette, IN, United States; ^2^School of Natural and Environmental Sciences, Newcastle University, Newcastle Upon Tyne, United Kingdom

**Keywords:** turkey, accelerometer, habituation, step count, gait, health status

## Abstract

Accelerometers have the potential to provide objective, non-invasive methods for detecting changes in animal behavior and health. Our objectives were to: (1) determine the effects of micro-acceleration data loggers (accelerometers) and habituation to accelerometers on turkey gait and health status, (2) determine age-related changes in gait and health status, and (3) assess the validity and reliability of the accelerometers. Thirty-six male commercial turkeys were randomly assigned to one of five groups: accelerometer and habituation period (AH), accelerometer and no habituation period (AN), VetRap bandage (no accelerometer) and habituation period (VH), bandage (no accelerometer) and no habituation period (VN), and nothing on either leg (C). Health status and body condition were assessed prior to video-recording birds as they walked across a Tekscan® pressure pad at 8, 12, and 16 weeks to determine effects of treatment on number of steps, cadence, gait time, gait distance, gait velocity, impulse, gait cycle time, maximum force, peak vertical pressure, single support time, contact time, step length, step time, step velocity, stride length, total double support time, and duty factor. Accelerometer validity and reliability were determined by comparing the number of steps detected by the accelerometer to the number of steps determined from video recordings. Several age-related changes in turkey gait were found regardless of habituation including a slower cadence at 16 weeks, shorter gait distance at 8 weeks, and slower gait velocity at 16 weeks. When comparing bandaged vs. unbandaged limbs, both treatment and age-treatment interactions were found depending on the gait parameter. Accelerometer validity and reliability were affected by both age and treatment. False discovery rate increased, while accuracy and specificity decreased with age. Validity and reliability were lowest for non-habituated birds (AN and VN). Results demonstrated that micro-data loggers do not adversely affect turkey health status, but habituation to wearing accelerometers greatly affects accelerometer reliability and validity. Accelerometer validity and turkey gait are also greatly affected by the age of the turkeys.

## Introduction

The use of wearable sensors is an emerging area of research in the animal behavior and welfare field. The ability to take objective, non-invasive measurements of the behavior of an animal is advantageous compared to visual inspection, which is most frequently used to evaluate animal behavior and health status. However, on large farms where thousands of birds are housed together, visual inspection can potentially lead to oversight because it is difficult to detect problems in individual animals among thousands. Moreover, visual inspection can only identify an issue after welfare has already been compromised (1, 2).

Wearable sensors have been applied to a variety of species for automated monitoring of animal health and behavior in research. Wearable sensors can record a variety of measures such as internal body temperature, environmental temperature, acceleration, heart rate, and step counts depending on the type used and where on the body it is placed (3). In particular, accelerometers can be a tool to define and record energy expenditure (4, 5), posture (6), and locomotor levels (7, 8) that can be early indicators of welfare concerns. Furthermore, acceleration output from activity level sensors can be used to distinguish between different behaviors performed by an animal. For example, accelerometers attached to a collar can accurately distinguish between grazing, ruminating, and resting behaviors of sheep (9). Attached to the leg, accelerometers show potential as an early indicator of lameness in laying hens (10), sheep (11), dairy cattle (12, 13), and horses (14). Behaviors associated with health status are also detectable using accelerometers or bio-loggers in Pekin ducks to detect lethargy caused by an immune challenge (15) and in laying hens as an early detector of avian influenza by assessing decreasing activity levels (16). Based on the aforementioned research, accelerometers are useful tools for the automatic, non-invasive monitoring of animal behavior, but limited research has been conducted to evaluate the use of accelerometers to monitor the behavior of turkeys. One study evaluated the validity and feasibility of using HOBO Pendant® (HPD) loggers for detecting steps of grower turkeys (9–11 weeks of age) and finisher turkeys (14 weeks) (17). Their results indicated that HPD loggers are capable of detecting step counts in turkeys. However, the HPD loggers are large (18 g in weight and 58(h) × 33(l) × 28(w) mm), making them cumbersome for measuring activity levels of young turkeys and therefore not suitable for detecting long-term changes in activity levels of growing turkeys. Furthermore, the HPD loggers were only able to record continuously for 54 min. Similarly to the HPD loggers, the study determining lethargy in Pekin ducks used a similarly sized sensor (Actical) at 17.5 g (15) while the laying hen study used a prototype accelerometer that is not commercially available (16). With recent advances in technology, it is now possible to use micro-data loggers that are much smaller, lighter, and that have a longer memory and battery capacity, enabling changes in animals' activity levels and number of steps taken to be recorded for longer periods of time and for smaller animals. However, no studies have evaluated the feasibility and reliability of using micro-data loggers for measuring activity levels of turkeys.

In addition to the size and weight of the accelerometer, the effect of the accelerometer on animal behavior is another important consideration. The presence of the accelerometer itself can cause changes in an animal's behavior. Habituation is an important concept when introducing novel technology to an animal (18, 19). Introducing a novel object can cause fear and affect the validity and reliability of a study (18). Furthermore, the presence of a sensor may cause the animal wearing that sensor to be targeted by pen-mates, leading to further changes in typical behavior and potential changes in animal health status. Due to the unfamiliar feeling of wearing an accelerometer, birds may also favor the leg with an accelerometer by applying less body weight on that foot (potentially affecting normal walking gait). Image-related sensors have shown potential as an alternative to accelerometers in terms of detecting bird movement (20). In many situations, image sensors can be more practical than accelerometers, however accelerometers have the potential to detect certain aspects image sensors currently cannot. For example overhead cameras can only detect movements from the top of the bird as the legs will not be seen in the camera's vision, while accelerometers can be attached to certain body parts of birds to target certain aspects of movement.

Age related changes in turkey gait, although not heavily researched, should be expected regardless of the effect of wearing an accelerometer. A few studies have documented a decrease in overall leg and footpad health in turkeys as they age, and we expect gait to also change with age (21–23). Krautwald-Junghanns et al. observed increasing severity in footpad dermatitis and lesions from 6 to 16 weeks of age (22). Dalton et al. (23) observed a worsening gait score as birds aged, but there has been no further research into the gait dynamics of these worsening scores (23). Turkeys have gone through extensive selective breeding in order to generate a fast-growing bird with a large breast muscle. These changes in body conformation can have effects on how turkeys walk, but limited research has investigated changes in turkey gait. Recently, Kremer et al. demonstrated that as female turkeys age, certain gait parameters such gait velocity, peak vertical force, and step length increased with age while other parameters such as gait cycle time were not affected (24). Similarly, Oviedo-Rondón et al. demonstrated that in male turkeys, certain gait dynamic change due to leg health and age. Step length was longer in birds without leg abnormalities, peak vertical force and impulse increased as a bird aged, and bipedal cycle time was affected by both leg health and age (25). These age-related studies indicate that both age and leg health play crucial roles in turkey gait dynamics, so introducing an unfamiliar accelerometer on the leg may further complicate how a bird walks. Male vs. female gait changes may also be a factor as hens displayed a longer step length with age, while males showed no change (24, 25).

The overall goal of this research was to evaluate the feasibility of using micro acceleration data loggers (accelerometers) for detecting steps and changes in activity levels of turkeys at different ages. Specific objectives included: (1) determining the effects of accelerometers and habituation to accelerometers on turkey gait, (2) determining age-related changes in gait, and (3) assessing the validity and reliability of the accelerometers.

## Materials and Methods

### Ethics Statement

This study was carried out in accordance with the recommendations and approval of the Institutional Animal Care and Use Committee of Purdue University.

### Experimental Procedures

A total of 44 beak-trimmed tom turkeys (Nicolas Select, Aviagen Turkeys, Lewisburg, West Virginia, USA) were obtained from a commercial hatchery at 1 d of age and housed at the Purdue Animal Sciences Research and Education Center (ASREC). From 1 to 7 d of age, the poults were housed together in a brooding ring, and then randomly assigned to 8 littered (wood shavings) pens (measuring 2.44 m by 1.52 m) with either 5 or 6 birds per pen. Each pen was supplied with a hanging feeder and bell drinker, providing feed and water *ad libitum*. Lighting and temperature were maintained according to Aviagen-recommended industry standards (26). For the first day, poults were provided with 24 h of light, which was gradually adjusted to a final photoperiod of 15 h light: 9 h of darkness by the fourth day. A minimum light intensity of 40 lux was provided. Room temperature was changed weekly as recommended by Aviagen (26). Briefly, poults were brooded at a temperature of 30°C, which was gradually decreased to a final temperature of 13°C at 14 weeks.

Birds were randomly assigned to one of five groups. Groups differed depending on whether they were habituated to wearing a VetRap™ bandage (with or without an accelerometer) for one week prior to data collection:

Habituated groups (H):

1. AH group: habituated to wearing both a bandage and an accelerometer. Habituation to the Tekscan occurred for 2 weeks and habituation to the bandage for 1 week prior to each data collection at 8, 12, and 16 weeks (*n* = 7).
2. VH group: habituated to wearing only a bandage. The accelerometer was attached only while data were collected on the Tekscan pressure sensing walkway at 8, 12, and 16 weeks. Habituation occurred for 1 week prior to each data collection at 8, 12, and 16 weeks (*n* = 8).

Non-habituated groups (NH):

AN group: the bandage and accelerometer were attached only when data were collected on the Tekscan pressure sensing walkway at 8, 12, and 16 weeks. No habituation to the bandage occurred (*n* = 4).
VN group: the bandage was attached only when data were collected on the Tekscan pressure sensing walkway at 8, 12, and 16 weeks. No accelerometer was attached and no habituation to the bandage occurred (*n* = 10).

Control group (C):

C group: no bandage or accelerometer were attached at any time during the study (C, *n* = 6). No habituation to the bandage occurred.

Sample sizes varied due to the number of birds in each pen and due to mortality of 8 birds over the course of the study. Two turkeys were found dead at 8 d, one at 10 d and one at 14 d, before data collection had started. One turkey from the control (C) group was found dead at 10 weeks. One turkey (AN group) was euthanized at 16 weeks due to a broken wing. Two turkeys were euthanized due to lameness at 13 (AN group) and 14 (AH group) weeks, respectively. The two lame birds' gait data were not used in the analysis of the study at 12 weeks. Only complete data sets from 36 birds were used in the final analyses.

**Figure 1:**
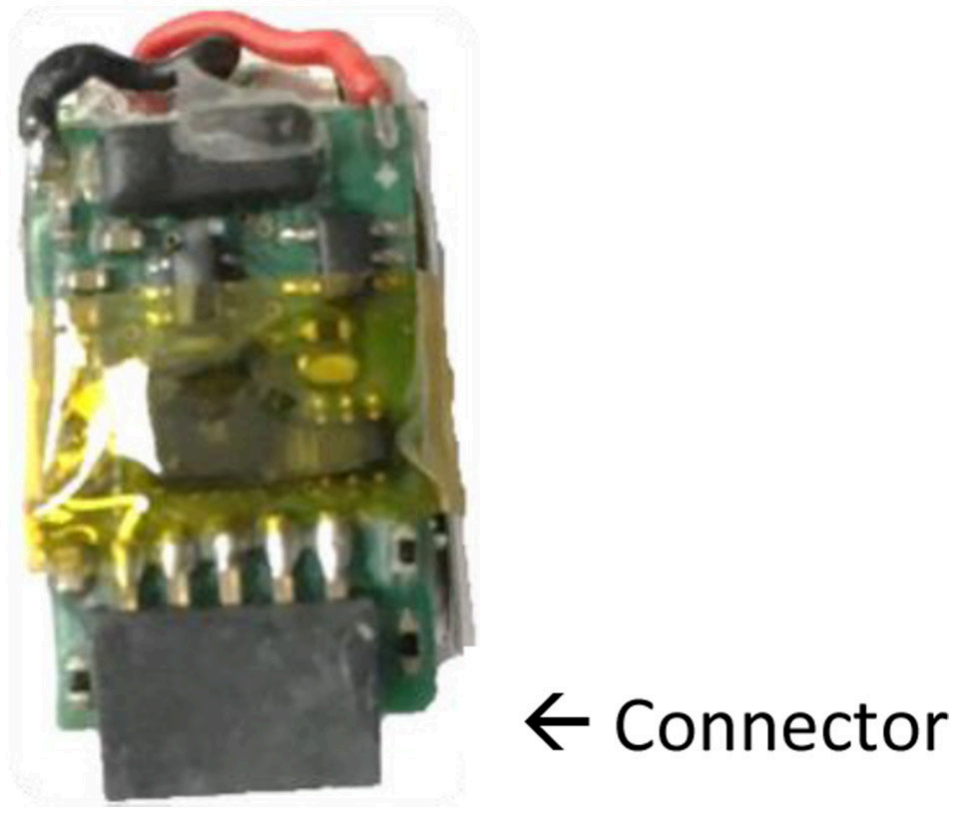
Orientation of the AXY-3 micro accelerometer.

**Figure 2:**
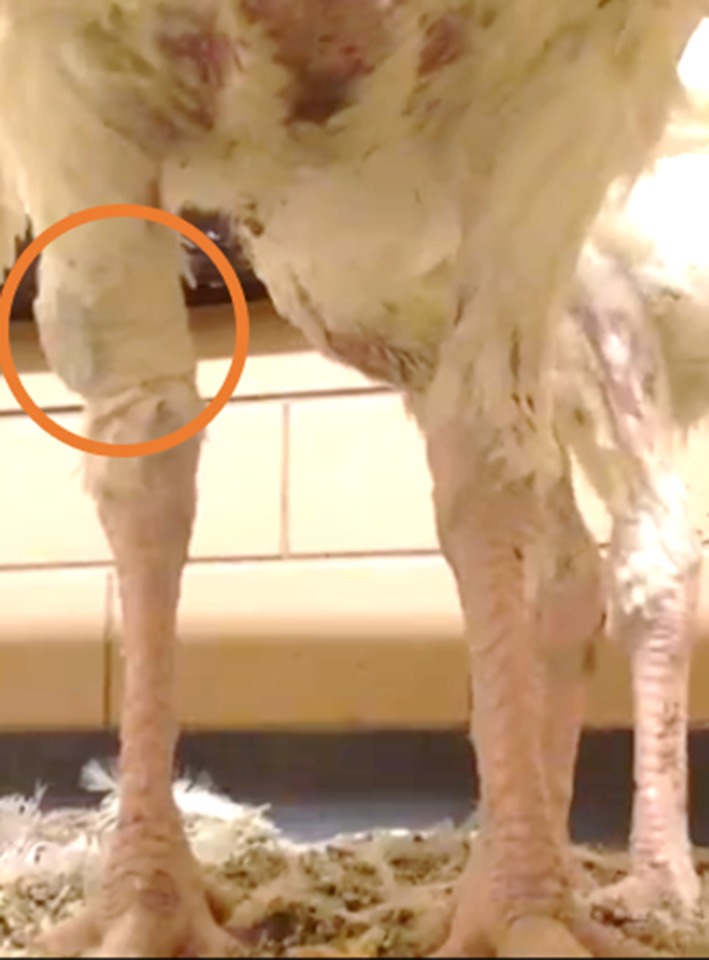
Location of AXY-3 micro accelerometer attached to the leg of the turkey.

**Figure 3:**
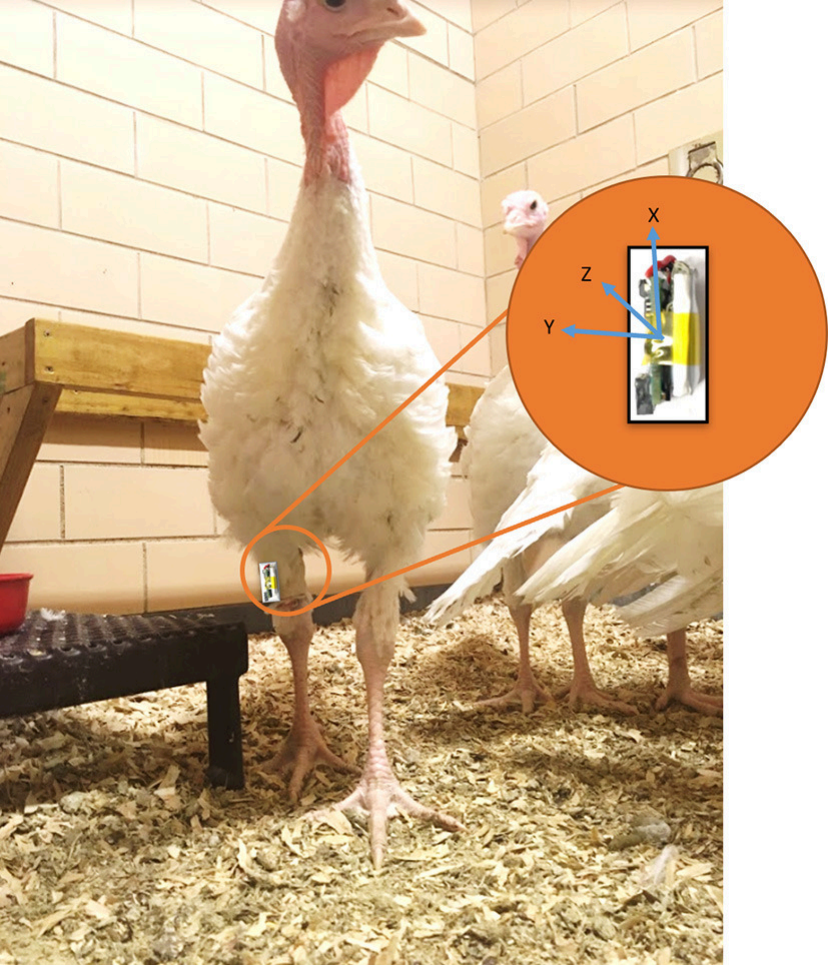
AXY-3 micro accelerometer axis orientation and placement on each bird.

In order to attach an accelerometer to a turkey's leg, a accelerometer (AXY-3 Micro Acceleration Data Loggers, TechnoSmArt, Guidonia-Montecelio, Italy) was sealed between two pieces of VetRap bandage, and then secured around the bird's leg with more bandage. The accelerometer was placed just above the hock, facing outward with the connector pointed toward the ground and the battery in contact with the leg (Figures 1–3). Accelerometer attachment was balanced for left and right legs across treatment groups. We used a total of 10 accelerometers and set accelerometers to record at a frequency of 10 Hz. The accelerometers recorded acceleration measurements in 3 dimensions (X, Y, Z). The accelerometers are 9.5 (l) × 15 (h) × 4 (w) mm, weigh 0.7 g, and have the potential to record for up to 30 days on a single charged battery (40).

We examined the effects of the accelerometer on turkey gait parameters at 8, 12, and 16 weeks using a Tekscan® pressure sensing walkway (Tekscan Inc, South Boston, MA, USA) and by analyzing video recordings of turkeys as they walked across the Tekscan. Some Tekscan gait parameters are calculated by the Tekscan software using both limbs [number of steps (strike number), cadence, gait time, gait distance, and gait velocity], while other measures are calculated for each limb separately (impulse, gait cycle time, maximum force as a percentage of body weight, peak vertical pressure, single support time, stance time, step length, step time, step velocity, stride length and total double support time, among others). Descriptions of gait parameters are provided in Table 1. Gait parameters were selected for analysis based on previous leg heath studies with the Tekscan system in turkey hens (24) and Pekin ducks (30).

**Table 1:** Description of measurements analyzed on the Tekscan pressure sensing walkway [adapted from Tekscan Walkway™ User Manual (27)] and duty factor [determined using methods of Gatesy and Biewener (28); Paxton et al. (29) and Oviedo-Rondón et al. (25)].

**Tekscan measure**	**Definition**
Cadence (steps/min)	Number of steps taken per minute
Gait time (s)	Time from first contact with the walkway to the last contact with the walkway
Gait distance (cm)	The distance from the heel of the first stance to the heel of the last stance
Gait velocity (cm/s)	Gait distance divided by gait time
Impulse (%)	Amount of force exerted on the walkway over the entire walk (as a percentage of body weight)
Gait cycle time (s)	Average time from the first contact of a foot to the next contact of the same foot
Maximum force (%BW)	Maximum amount of pressure exerted onto the walkway as a percentage of the subject's body weight
Peak pressure (KPa)	The maximum pressure value recorded
Single support time (s)	The time that the foot is in contact with the walkway
Stance time (s)	The average time from when the foot first comes into contact with the walkway to the last time when the foot is in contact with the walkway
Step Length (cm)	The average distance from the heel of the first foot to the heel of the second foot in a single stride
Step time (s)	Elapsed time from the first contact of the foot to the walkway to the first contact of the opposite foot to the walkway
Step velocity (cm/s)	Step length divided by step time
Stride length (cm)	The distance between consecutive footprints of the same foot
Total double support time	A foot's initial double support time (time from first contact of the foot to last contact of the opposing foot's next stance) added to the same foot's terminal double support time (time of first contact of the opposing foot to last contact of the foot under consideration)
Contact time (s)	Total time a foot was in contact with the walkway
Duty factor (s)	Contact time/Gait cycle time; stride data derived from the point where the foot was in contact with the ground

The Tekscan was placed in the aisle between turkeys' pens. A runway was constructed to ensure that turkeys remained on the Tekscan pressure sensing walkway. The runway was the same length as the Tekscan (0.58 × 1.09 m) and consisted of a piece of clear plexiglass secured by two wooden support boards. Two clear plastic mats were also placed on the floor on either side of the Tekscan so that the birds would walk on the same type of surface to prevent changes in gait as the birds stepped on the Tekscan.

A video camera was positioned 1.23 m from both ends of the mat to record turkeys as they walked across the Tekscan. The camcorders (Sony Camcorders, CX405, Sony Corporation of America, New York, NY) were attached to a tripod at a height of approximately 0.61 m. On data collection days, VN, VH, AN, AH, and C birds were tested in random order. During the recording process, one researcher was positioned approximately 3 m from the center of the Tekscan to operate the laptop that controlled the Tekscan. Another researcher removed individual turkeys from their respective pens, applied the determined treatment to the leg, and positioned the bird in front of the first plastic mat. Birds were recorded as they walked across the Tekscan in one direction, then back across the Tekscan in the other direction. The bird would walk down the constructed walkway (pass 1), and then the researcher would walk to the other end of the Tekscan to have the bird take another pass across the walkway (pass 2). All birds had been habituated to this setup and process of walking with a researcher present prior to data collection.

Birds were habituated to the Tekscan for the 2 week period before each data collection. At 6 weeks, turkeys were placed on the Tekscan for 30 min daily for 2 weeks. However, the 30 min habituation period was shortened to 15 min for the 2 weeks prior to 12 and 16 weeks because birds began to rest 15 min into the habituation sessions. The habituation procedure included removing an entire pen of turkeys and re-locating the birds to the Tekscan set up. During the 15 min session, birds would be encouraged to walk over the Tekscan several times to get used to walking on the plastic surface.

Starting at 4 weeks of age, turkeys were marked every 2 weeks with black non-toxic livestock marker (Prima Tech Marking Stick, Neogen Corp., Lansing, MI, USA) for identification purposes and to ensure that markings remained visible. The health status and body condition (feather condition, footpad health, feather cleanliness, body condition, and body weight) of the turkeys were checked and recorded before each day of data collection at 8, 12, and 16 weeks. Body feather condition was scored as 0 (little to no missing or broken feathers), 1 (feather loss/damage up to 5 cm in diameter), or 2 (feather loss or damage of 5 cm or greater) [adapted from (31)]. Feather condition of the wings and tail were scored as 0 (no broken or missing feathers), 1 (< 25% missing or broken feathers), 2 (between 25 and 50% missing or broken feathers) or 3 (more than 50% missing or broken feathers). Feather cleanliness was scored as 0 (no soiling), 1 (moderately soiled) or 2 (severe soiling). Footpad health was scored according to the Global Animal Partnership standards for turkeys (2015) as 0 (no lesions, swelling, or erosion of the footpad), 1 (mild or superficial lesions and/or thickened skin), or 2 (severe lesions, ulcers and/or scabs).

### Statistical Analysis

#### Effects of Treatment and Age on Turkey Health, Body Condition and Gait

Age related changes in turkey health status and body condition measures were analyzed in SPSS (version 25) for H, C, and NH groups using a Friedman test with a *post hoc* Wilcoxon test and Bonferroni adjustment for multiple pairwise comparisons. Treatment related differences were analyzed in SPSS with a Kruskal-Wallis test. The majority of turkeys received scores of 0 for the various health and body condition measures. Therefore, statistical analyses comparing age and treatment effects were only performed on tail, left wing, and right wing feather condition scores.

Tekscan data were analyzed to determine the effects of age (8, 12, and 16 weeks) and treatment group (H, NH, and C) on gait parameters. Tekscan data were selected for analysis if several conditions were met:

1. All toes were present on the pressure pad at each step
2. The bird walked continuously during the walk, without stopping, standing, or jumping
3. There were at least 4 consecutive steps taken

In addition to the gait parameters derived from the Tekscan, we calculated the duty factor [derived from (25, 28, 29)] to incorporate previous avian gait dynamics known to change with age (24, 25, 28). Duty factor is a measure of the total stride cycle when the foot is in contact with the ground (Table 1).

Tekscan parameters and duty factor were analyzed using a repeated measures model (PROC MIXED, SAS 9.4) that included pen as a random effect. The following analyses were conducted:

1. Tekscan measures calculated taking both limbs into consideration (number of steps, cadence, gait time, gait distance and gait velocity): treatment, age and their interaction were included as factors to determine whether Tekscan parameters changed with age and due to habituation to the VetRap bandage.
2. Tekscan measures calculated for each limb (impulse, gait cycle time, maximum force (% BW), peak pressure, single support time, stance time, step length, step time, step velocity, stride length, and total double support time) and duty factor: treatment, age, limb (bandaged or not), and all their interactions were included in a model to evaluate whether there were differences between bandaged and unbandaged limbs within treatment groups, and between bandaged or unbandaged limbs among treatment groups and ages. In order to do these comparisons, we had determined that there were no differences between left and right limbs of C birds (PROC MIXED, SAS 9.4 with limb and age as factors, individual bird as the repeated measure and pen as a random effect). Consequently, we randomly assigned one limb of each C bird as the designated limb for comparison so that we could analyze differences between limbs of each treatment group.

#### Validity and Reliability of Accelerometers

**Figure 4:**
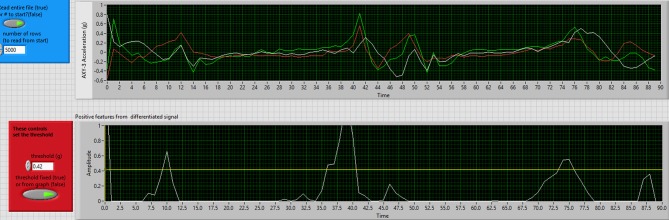
Accelerometer step detection output example after processing through LabVIEW. The top graph depicts the X, Y, and Z axis accelerations while the bottom graph depicts the processed acceleration data where all three axis have been combined. The yellow line on the bottom graph depicts the step threshold and any acceleration peaks above the threshold indicates that a bird is taking a step.

Validity and reliability of accelerometers were analyzed using data from VH, AH, and AN as these groups all had accelerometers attached at the time that birds were walking across the Tekscan walkway. In order to analyze data obtained from the accelerometers, the accelerometer output was transformed and smoothed in LabVIEW (National Instruments, Austin, Texas, USA) using an adapted Pan-Tompkins algorithm (32) based on the methods of Dalton et al. to determine the number of steps taken by individual birds (17). The Methods of Dalton et al. were modified to include all three axes (X, Y, and Z), which were combined into a single variable within the Lab VIEW program [Figure 4; 17]. To determine the number of steps taken, a step threshold was selected so any acceleration values above the step threshold were considered steps, whereas values below the threshold were not considered steps. The step threshold varied depending on the age of the birds and was set at 0.42 g/s for 8 weeks, 0.53 g/s for 12 weeks, and 0.66 g/s for 16 weeks (where g represents acceleration due to gravity). The step threshold was examined every 0.01 g/s between the range of 0.3–0.8 g/s and the step threshold level was set to when the cumulative sensitivity for each age group was highest (17, 33).

In this study, a step was defined as the point when all toes of one foot were off the ground while the footpad of the other foot remained in contact with the ground. Video camera step counts were determined by visually counting steps every 0.1 s as the bird walked across the Tekscan. A scoring system of 0 and 1 was used to score when the bird took a step (1) or was not stepping (0) [as described in (8, 17)]. A single observer (RS) conducted video analyses to determine when the bird did and did not take a step. The accelerometer step count was compared to the step count determined from video recordings to calculate the sensitivity, accuracy, false discovery rate, specificity and precision of the accelerometers (17). The accelerometer and video data were both synchronized using a watch and an audio cue on the video so that the researcher verbally stated the moment and the time at which the accelerometer was activated, thus enabling the video time stamp to be matched with the accelerometer time stamp. A true positive was the number of steps detected by the accelerometer that were also observed on the video recording, whereas a true negative was the number of non-stepping time points detected by the accelerometer that were also non-stepping time points on the video recording. A false positive occurred when the accelerometer detected a step, but no step was observed on the video recording, while a false negative occurred when the video determined the bird was stepping, but no step was detected by the accelerometer. The following equations were utilized to determine sensitivity, accuracy, false discovery rate, specificity, precision, and cumulative sensitivity of the accelerometers (17):

$$\begin{array}{l} Sensitivity\;=\;True\;Positive\;/\;(True\;Positive\;\\ \;+\;False\;Negative)\times \;100\;\\ \;Accuracy\;=\;(True\;Positive\text{ ​}+\text{ ​}True\;Negative)\;/\;\\ \;(True\;Positive+True\;Negative\;\\ \;False\;Positive+\text{​}False\;Negative)\text{ ​}\times \text{ ​}100\\ False\;discovery\;rate\;=\;False\;Positive\;/\;(False\;Positive\;\\ \;+\;True\;Positive)\;\times \;100\;\\ \;Specificity\;=\;True\;Negative\;/\;(True\;Negative\;\\ \;+\;False\;Positive)\;\times \;100\;\\ \;Precision\;=\;True\;Positive\;/\;(True\;Positive\;\\ \;+False\;Positive)\;\times \;100\;\\ Cumulative\;Sensitivity=Sensitivity-False\;Discovery\;Rate \end{array}$$

## Results

### Treatment and Age Effects on Turkey Health Status and Body Condition

As expected, average body weight ± sd increased with age (8 weeks: 3.81 ± 0.34 kg, 12 weeks: 9.24 ± 0.79 kg and 16 weeks: 15.17 ± 1.20 kg), but did not differ among treatment groups. Snood wounds were noted on three birds at 12 weeks (one each from AN, VH, and C with two birds being from the same pen). The large majority of the scores for footpad health, feather cleanliness, and feather condition of the neck, rump, and back were 0, so only tail and wing feather scores were included in further analysis. Wing and tail feather condition scores were not significantly different between VH and AH or between VN and AN groups; therefore to increase power of the results, treatment groups were combined into habituated (AH, VH; *n* = 17), non-habituated (AN, VN; *n* = 10), and control (C; *n* = 8) groups to analyze age differences. Both wing and tail feather condition varied due to age for all treatment groups. The habituated (H) birds had lower tail feather scores at 8 weeks (median; 25th quartile, 75th quartile: 0; 0, 0.5) compared to scores at 12 weeks (1; 1, 1) and 16 weeks (1; 1, 1; *P* < 0.0001). Feather damage was worse for both left and right wings at 12 weeks (2; 2, 2) compared to 8 weeks (1; 1,1) and 16 weeks (1; 1, 1; *P* < 0.0001). Similar to H turkeys, tail feather scores of NH turkeys were less severe at 8 weeks (1; 0, 1) compared to 12 weeks (1; 1, 1) and 16 weeks (1; 1, 1; *P* = 0.018). Wing feather damage was also more severe at 12 weeks (left wing: 2; 1.75, 2; right wing: 2; 1, 2) compared to 8 weeks (left wing: 1; 1, 1; right wing: 1; 1, 1; *P* < 0.01) and 16 weeks (left wing: 1; 1, 1); right wing: 1; 1, 1; *P* < 0.01). Turkeys in the C group had the least tail feather damage at 8 weeks (0; 0, 1) compared to 12 weeks (1; 1, 1) and 16 weeks (1, 1,1; *P* = 0.007). Wing feather damage was worst at 12 weeks (2, 1, 2) compared to both 8 weeks (1; 1,2) and 16 weeks (1; 1, 2; *P* = 0.039).

### Treatment and Age Effects on Turkey Gait

#### Tekscan Measures Calculated Using Both Limbs

Tekscan gait parameter results are presented in Table 2. There was a significant interaction for age and treatment for the number of steps taken, with *post hoc* comparisons indicating a tendency for the number of steps to be higher for H turkeys than C turkeys at 8 weeks (*P* = 0.06). No other significant differences were found. Cadence varied due to age, with cadence being higher at 16 weeks compared to 8 and 12 weeks, and higher at 12 weeks compared to 8 weeks (Table 2). No significant effects of age, treatment or their interaction were found for gait time. Gait distance was significantly longer at 12 weeks compared to 8 weeks, but no other significant differences were found. Similarly, gait velocity varied with age, and was faster at 12 weeks than at 16 weeks (*P* = 0.01), but no other significant differences were found.

**Table 2:** Differences in least square means (± standard error) Tekscan gait parameters^*^ among turkeys habituated to wearing a VetRap bandage with or without an attached accelerometer (H group), turkeys not habituated to wearing a bandage or accelerometer (NH group) and control turkeys that did not wear any bandage or accelerometer at any time (C group) at 8, 12, and 16 weeks of age.

**Gait parameter**	**Treatment group**	**Age**			**Main effect (treatment)**
		**8 weeks**	**12 weeks**	**16 weeks**
Number of steps	H	7.55 ± 0.47	6.93 ± 0.49	7.55 ± 0.49	7.34 ± 0.30
	NH	7.29 ± 0.53	7.53 ± 0.53	6.35 ± 0.53	7.10 ± 0.33
	C	5.40 ± 0.53	7.00 ± 0.59	7.45 ± 0.56	6.62 ± 0.35
Main effect (age)		6.75 ± 0.31	7.15 ± 0.32	7.12 ± 0.32	
Cadence	H	92.25 ± 6.95	86.21 ± 6.46	70.68 ± 6.46	83.05 ± 4.35
	NH	92.25 ± 6.26	77.73 ± 6.95	73.38 ± 7.01	77.89 ± 4.66
	C	78.28 ± 6.95	85.83 ± 7.65	66.18 ± 7.29	76.76 ± 4.79
Main effect (age)		84.37 ± 4.60^a^	83.26 ± 4.72^b^	70.07 ± 4.66^c^	
Gait time	H	4.08 ± 0.47	3.88 ± 0.49	5.15 ± 0.49	4.37 ± 0.31
	NH	4.33 ± 0.54	4.63 ± 0.53	3.93 ± 0.53	4.30 ± 0.34
	C	3.70 ± 0.53	3.63 ± 0.59	5.37 ± 0.56	4.24 ± 0.35
Main effect (age)		4.04 ± 0.34	4.05 ± 0.35	4.82 ± 0.34	
Gait distance	H	113.63 ± 7.60^b^	122.05 ± 7.90^a^	123.63 ± 7.90^a^	119.77 ± 4.77
	NH	113.64 ± 8.65^b^	126.43 ± 8.62^a^	108.10 ± 8.65^a^	116.05 ± 5.24
	C	83.09 ± 8.63^b^	127.67 ± 9.62^a^	113.60 ± 9.09^a^	108.12 ± 5.54
Main effect (age)		103.45 ± 4.96^b^	125.38 ± 5.19^a^	115.11 ± 5.09^a, b^	
Gait velocity	H	30.64 ± 3.09^b^	35.74 ± 3.17^a^	25.82 ± 317^a^	30.73 ± 2.03
	NH	28.33 ± 3.08^b^	29.04 ± 3.39^a^	28.24 ± 3.42^b^	28.54 ± 2.19
	C	27.64 ± 3.42^b^	35.94 ± 3.71^a^	22.96 ± 3.56^a^	28.85 ± 2.17
Main effect (age)		28.87 ± 2.32^a, b^	33.57 ± 2.37^a^	25.67 ± 2.34^b^	

a, b, c *Means within rows that have different letters are significantly different (P < 0.05)*.

* *These Tekscan measures are provided by the Tekscan software as a single value (not for each foot independently of the other foot)*.

In order to determine whether the presence of a bandage affected turkey gait, we compared gait parameters among bandaged and unbandaged limbs at each age and for each treatment group. Limbs from control birds were randomly designated as a “bandaged” limb in order to compare gait parameters among treatment groups; previous analyses had indicated that there were no differences in any gait parameters between left and right limbs of C birds. There were no significant differences for any gait parameters between bandaged and unbandaged limbs. Therefore, limb parameters were combined and we report the effects of age, treatment group and their interaction on gait parameters in Table 3.

**Table 3:** Differences in least square means (± standard error) Tekscan gait parameters^*^ among turkeys habituated to wearing a VetRap bandage with or without an attached accelerometer (H group), turkeys not habituated to wearing a bandage or accelerometer (NH group) and control turkeys that did not wear any bandage or accelerometer at any time (C group) at 8, 12, and 16 weeks of age.

**Gait parameter**	**Treatment group**	**Age**			**Main effect (treatment)**
		**8 weeks**	**12 weeks**	**16 weeks**
Impulse	H	56.76 ± 4.98^b^	72.57 ± 5.01^a^^,^^b^	82.21 ± 5.26^a^	70.51 ± 3.76
	NH	71.37 ± 5.93	80.62 ± 6.15	77.09 ± 5.65	76.36 ± 4.15
	C	68.58 ± 5.60^b^	70.0 ± 6.12^a, b^	91.75 ± 5.85^a^	76.78 ± 4.13
Main effect (age)		65.60 ± 3.96^b^	74.40 ± 4.07^a, b^	83.68 ± 3.98^a^	
Gait cycle time	H	1.35 ± 0.12^b^	1.42 ± 0.12^b^	1.91 ± 0.13^a^	1.56 ± 0.10
	NH	1.60 ± 0.15	1.75 ± 0.15	1.64 ± 0.14	1.67 ± 0.11
	C	1.68 ± 0.14	1.42 ± 0.15	1.88 ± 0.15	1.66 ± 0.11
Main effect (age)		1.54 ± 0.10^b^	1.53 ± 0.10^b^	1.81 ± 0.10^a^	
Maximum force (% body weight)	H	97.53 ± 2.36	112.36 ± 2.38	106.63 ± 2.47	105.50 ± 2.05
	NH	97.02 ± 2.98	108.23 ± 2.91	102.56 ± 2.70	102.61 ± 2.26
	C	97.41 ± 2.88	105.40 ± 3.06	101.23 ± 2.97	101.35 ± 2.57
Main effect (age)		97.32 ± 1.55^c^	108.66 ± 1.62^a^	103.47 ± 1.57^b^	
Peak pressure	H	83.73 ± 3.59	147.75 ± 3.63	163.53 ± 3.82	131.19 ± 3.15
	NH	86.60 ± 4.37	144.16 ± 4.63	162.82 ± 4.19	131.67 ± 2.78
	C	96.50 ± 4.29	146.83 ± 4.70	169.24 ± 4.49	137.52 ± 3.45
Main effect (age)		88.94 ± 2.37^c^	146.24 ± 2.51^b^	165.20 ± 2.40^a^	
Single support time	H	0.42 ± 0.03	0.33 ± 0.03	0.34 ± 0.03	0.36 ± 0.02
	NH	0.48 ± 0.04	0.38 ± 0.04	0.34 ± 0.03	0.40 ± 0.02
	C	0.45 ± 0.03	0.33 ± 0.04	0.36 ± 0.04	0.38 ± 0.02
Main effect (age)		0.45 ± 0.02^a^	0.34 ± 0.02^b^	0.35 ± 0.02^b^	
Stance time	H	0.95 ± 0.10	1.07 ± 0.01	1.36 ± 0.10	1.13 ± 0.08
	NH	1.22 ± 0.11	1.21 ± 0.12	1.21 ± 0.11	1.22 ± 0.08
	C	1.19 ± 0.11^b^	1.13 ± 0.12^b^	1.58 ± 0.11^a^	1.30 ± 0.09
Main effect (age)		1.12 ± 0.08^b^	1.14 ± 0.08^b^	1.68 ± 0.08^a^	
Step length	H	19.75 ± 0.68^c^	24.30 ± 0.68^a^	22.05 ± 0.73^b^	21.91 ± 0.40
	NH	19.69 ± 0.84	23.02 ± 0.90	23.02 ± 0.80	22.03 ± 0.39
	C	20.39 ± 0.80^b^	24.71 ± 0.89^a^	20.78 ± 0.84^a, b^	21.96 ± 0.55
Main effect (age)		19.94 ± 0.45^c^	24.01 ± 0.48^a^	21.95 ± 0.46^b^	
Step time	H	0.68 ± 0.06	0.73 ± 0.06	0.90 ± 0.07	0.77 ± 0.05
	NH	0.83 ± 0.08	0.87 ± 0.08	0.80 ± 0.07	0.83 ± 0.05
	C	0.88 ± 0.07	0.71 ± 0.08	0.95 ± 0.08	0.85 ± 0.05
Main effect (age)		0.80 ± 0.05	0.77 ± 0.05	0.88 ± 0.05	
Step velocity	H	32.62 ± 2.79	37.09 ± 2.82	27.17 ± 2.96	32.39 ± 2.18
	NH	30.15 ± 3.35	29.78 ± 3.48	31.36 ± 3.19	30.43 ± 2.41
	C	28.63 ± 3.18^a, b^	36.48 ± 3.47^a^	22.73 ± 3.32^b^	29.28 ± 2.47
Main effect (age)		30.47 ± 2.16^a, b^	34.45 ± 2.23^a^	27.09 ± 2.17^b^	
Stride length	H	38.82 ± 1.34^b^	48.40 ± 1.35^a^	43.48 ± 1.43^a, b^	43.57 ± 1.00
	NH	38.57 ± 1.64^b^	44.73 ± 1.74^a, b^	45.05 ± 1.57^a^	42.78 ± 1.14
	C	40.44 ± 1.59^b^	48.55 ± 1.75^a^	41.85 ± 1.67^b^	43.61 ± 1.23
Main effect (age)		39.28 ± 0.89^c^	47.22 ± 0.95^a^	43.46 ± 0.91^b^	
Total double support time	H	0.54 ± 0.08^b^^,^ ^Y^	0.74 ± 0.08^b^	1.08 ± 0.08^a^	0.79 ± 0.06^X, Y^
	NH	0.49 ± 0.10^b^^,^^Y^	0.84 ± 0.10	0.85 ± 0.09^a^^,^^Y^	0.73 ± 0.07^Y^
	C	0.87 ± 0.09^b^^,^ ^X^	0.79 ± 0.10^b^	1.23 ± 0.09^a^^,^ ^X^	0.97 ± 0.07^X^
Main effect (age)		0.64 ± 0.06^c^	0.79 ± 0.07^b^	1.06 ± 0.06^a^	
Duty factor	H	0.71 ± 0.04	0.75 ± 0.04	0.73 ± 0.04	0.73 ± 0.02
	NH	0.78 ± 0.04	0.71 ± 0.05	0.76 ± 0.04	0.75 ± 0.03
	C	0.72 ± 0.04	0.77 ± 0.05	0.83 ± 0.04	0.77 ± 0.03
Main effect (age)		0.73 ± 0.03	0.74 ± 0.03	0.77 ± 0.03	

a, b, c *Means within rows that have different letters are significantly different (P < 0.05)*.

X, Y *Means within columns that have different letters are significantly different (P < 0.05)*.

* *These Tekscan measures are provided by the Tekscan software for each foot separately, but because feet did not differ from one another, we report least square means for both feet combined*.

The only significant treatment effect that was found was for total double support time, which was longer for NH birds than for C birds, with H birds being intermediate but not different from the other two groups at both 8 and 16 weeks. All gait parameters, excluding step time and duty factor varied with age (Table 3). Impulse was higher at 16 weeks than at 8 weeks, whereas gait cycle time and stance time were higher at 16 weeks than at 8 and 12 weeks. Maximum force (as a percentage of body weight), peak pressure, stride length, and total double support time differed among all ages, with peak pressure and total double support time being highest at 16 weeks. However, maximum force, step length and stride length were highest at 12 weeks. Step velocity was lower at 16 weeks than at 12 weeks, with step velocity at 8 weeks being intermediate.

The interaction between age and treatment was significant for impulse, gait cycle time, stance time, step velocity, stride length, and total double support time. Impulse of H and C birds was higher at 16 weeks than at 8 weeks. Gait cycle time was longer for H birds at 16 weeks than at 8 and 12 weeks. Stance time of C birds was longer at 16 weeks compared to 8 and 12 weeks. Step velocity of C birds was higher at 12 weeks than at 16 weeks, with step velocity at 8 weeks being intermediate. Stride length of H birds was longer at 12 weeks compared to 8 weeks, with stride length at 16 weeks being intermediate. Similarly, stride length of C birds was higher at 12 weeks compared to both 8 and 16 weeks. The only significant treatment effect that was found was for total double support time, which was longer for NH birds than for C birds, with H birds being intermediate but not different from the other two groups at both 8 and 16 weeks (*P* < 0.001). At 8 weeks, turkeys in the C group walked with a higher total double support time compared to both H and NH groups (*P* = 0.001). At 16 weeks, turkeys in the C and H group had higher total double support times compared to NH turkeys (*P* = 0.03). Total double support time of H and C birds were higher at 16 weeks than at 8 and 12 weeks (*P* = 0.01); NH birds had a lower total double support time at 8 weeks compared to 12 and 16 weeks (*P* = 0.02).

### Accelerometer Reliability and Validity

The sensitivity, accuracy, false discovery rate, specificity, and precision of the accelerometers were calculated by comparing the number of steps determined from the accelerometers to the number of steps determined from video recordings (Table 4). Accelerometers detected fewer steps and had false positives and negatives compared to video observations (Tables 4, 5). Age and treatment affected accelerometer precision (age only), sensitivity (age only), accuracy (age and treatment), specificity (age and treatment), and false discovery rate (age and treatment) (Table 5). No age-treatment interactions were found for any of the accelerometer parameters.

**Table 4:** Total number of steps recorded from video and accelerometers for the three accelerometer-wearing treatment groups^1^.

**Treatment**	**Video step counts (Total)**	**Accelerometer step counts (Total)**	**True positive**	**True negative**	**False positive**	**False negative**
AH	89	87	84	82	3	14
AN	69	86	59	58	35	10
VH	116	107	101	89	6	15

*True positive, true negative, false positive, and false negative were determined from comparing accelerometer output to the true number of steps from the video output*.

1 *Birds were assigned to treatment groups: AH, habituated to wearing an accelerometer; AN, not habituated, but wearing an accelerometer; VH, habituated to wearing a bandage*.

**Table 5:** Mean (± standard error) false discovery rate, sensitivity, accuracy, specificity and precision of the accelerometers relative to video observations of step counts at 8, 12, and 16 weeks for each treatment group^1^.

**Variable**	**Treatment**	**8 weeks**	**12 weeks**	**16 weeks**
Precision	AH	84.54 ± 0.50^a, b^	85.48 ± 0.54^a^	83.25 ± 0.49^b^
	AN	85.73 ± 0.56^a^	85.94 ± 0.52^a^	78.19 ± 0.52^b^
	VH	86.18 ± 0.47^a^	85.40 ± 0.45^a^	80.44 ± 0.46^b^
Sensitivity	AH	88.53 ± 0.52^a^	87.49 ± 0.56^a^	81.72 ± 0.55^b^
	AN	88.93 ± 0.60^a^	87.41 ± 0.56^a^	82.14 ± 0.55^b^
	VH	88.36 ± 0.49^a^	86.54 ± 0.49^a^	82.84 ± 0.52^b^
Accuracy	AH	88.55 ± 0.26^a^	88.02 ± 0.28^a^	84.46 ± 0.28^b^
	AN	84.65 ± 0.30^b^	83.73 ± 0.28^b^	84.60 ± 0.28^a, b^
	VH	87.98 ± 0.25^a^	87.29 ± 0.25^a^	84.43 ± 0.26^b^
Specificity	AH	84.00 ± 0.24^a^	85.72 ± 0.28^a, b^	82.15 ± 0.27^c^
	AN	84.30 ± 0.28^a^	83.19 ± 0.26^a^	79.42 ± 0.26^b^
	VH	83.86 ± 0.23^a, c^	86.38 ± 0.23^b^	82.17 ± 0.24^c^
False Discovery Rate	AH	11.69 ± 0.47^a^	11.45 ± 0.50^a, b^	21.18 ± 0.50^c^
	AN	15.34 ± 0.54^b^	10.73 ± 0.50^b^	25.35 ± 0.50^d^
	VH	12.08 ± 0.44^a^	12.10 ± 0.44^a^	22.00 ± 0.47^c^

a, b, c, d *Different means within each variable differ statistically (P < 0.05)*.

1 *Birds were assigned to treatment groups: AH, habituated to wearing an accelerometer*;

*AN, not habituated, but wearing an accelerometer*.

## Discussion

We examined changes in bird health status and body condition immediately prior to data collection at 8, 12, and 16 weeks to determine any changes due to age or treatment and to assess whether a bird was healthy to include in the gait analysis. No differences in health status and body condition due to treatment were detected. Therefore, we can infer that despite the changes in gait due to wearing an unfamiliar accelerometer, no negative side effects were observed in terms of feather condition, body weight, feather cleanliness, or footpad health. When accelerometers were first placed on each bird, birds pecked at the VetRap intermittently for several minutes (we did not systematically collect data to examine this behavior). Some birds were also observed to shake and kick the leg that had the bandage attached to it, but this behavior was only seen when the bandage was first applied. There were two instances of the birds successfully tearing off the bandage during a habituation period at 12 weeks and then another at 16 weeks. The bandage and accelerometer were then re-applied the same day, and accelerometers did not appear to shift during any other incidents. The other pen mates did not appear to be interested in the birds' leg that had a bandage applied, and we did not observe instances of other birds in the pen pecking at bandages. No long term behavioral or health issues were observed by the researchers. In terms of health changes due to age, the feather condition of both left and right wing feathers peaked in severity at 12 weeks, indicating that feather damage from feather pecking was highest at 12 weeks of age. Tail feathers had the highest scores at both 12 and 16 weeks of age. The welfare scores were to be expected as several studies have outlined an increase in injurious feather pecking as a turkey ages (23, 34, 35).

As a turkey ages, gait variables would be expected to change due to physical and morphological changes, such as increased body weight and leg length. In addition, overall foot and leg health have been shown to decline with age in domestic turkeys, resulting in poorer gait scores in older birds (21–23). There were several variables for which age affected how a bird walked including cadence, gait distance, and gait velocity while number of steps and gait time remained unchanged (Table 2). Results of this study indicated that by the time turkeys reach 16 weeks of age, gait parameters change such that turkeys take fewer steps per minute (cadence), spend more time on both feet (single and total double support time) and exert more pressure on the ground (peak pressure and maximum force) than at 8 and 12 weeks of age. At 12 weeks of age, several gait parameters peaked compared to 8 and 16 weeks. Generally, turkeys walked faster (gait velocity) and took longer steps (step length and stride length) at 12 weeks than at 8 weeks and 16 weeks. Some of the age-related changes reported here have not been observed in other studies. Cadence is the number of steps taken per minute (27), which was lower at 16 weeks of age compared to 8 and 12 weeks. Cadence had not been previously shown to decrease with age in turkey hens (24). Differences due to turkey sex may be a factor as hens displayed a longer step length with age (24), while no changes were observed for male turkeys (25). It is possible that continued exposure to the Tekscan over time resulted in a lower cadence by 16 weeks in our study. Alternatively, the birds could have walked at a slower pace due to an increase in body size or decline in leg health. We also observed a longer gait distance and slower gait velocity at both 12 and 16 weeks of age further confirming that cadence would also be lower if birds are taking a slower and longer stride (Table 2). Although Kremer et al. (24) did not see a significant difference in cadence as turkey hens age, they did see a slower gait velocity with age, similar to our findings and those of Oviedo-Rondon et al. (25). Oviedo-Rondon et al. (25) also reported that step length of male turkeys increased with age (13, 15, and 20 weeks), which is similar to the results of our study; however, step length was at its highest at 12 weeks and then decreased at 16 weeks (25).

Using the gait parameters that compared bandaged vs. unbandaged limbs, almost all were affected by age (Table 3). More specifically, total double support time, stride length, step velocity, step length, stance time, single support time, peak vertical pressure, maximum force, gait cycle time, and impulse were all found to have changes associated with age. Although it was initially, anecdotally observed that non-habituated birds displayed behaviors of discomfort, such as kicking or pecking at the bandage, many gait parameters seemed unaffected by treatment group. A possible explanation is that heavier birds are unable to maintain balance using a longer stride relative to leg length unlike younger, lighter birds. The overall gait dynamic of swing could also have changed causing more medial-lateral swing rather than a straight-line path (indicated by the changes in double support time, single support time, and gait time changes). Previous research comparing broiler and laying hen gait demonstrated that laying hens walk a more straight-line path while broilers possess greater body movements (36). It may be that turkeys, more similar to broilers, have more body oscillations compared to laying hens. However, unlike broilers, turkeys had a longer step as they aged due to a greater increase in leg length. Similar to Kremer et al. single support time (main effects) decreased with age in our study (24). It seems that most speed related parameters tend to decrease as turkeys get older after peaking at a certain age. Surprisingly, duty factor was unchanged throughout the study. In male turkeys it had been found that duty factor decreases with age much like the other gait parameters analyzed, however there is an inconsistency as Kremer et al. reported an overall decrease in duty factor with turkey hens (24). Oviedo-Rondón et al.'s male turkey study analyzed gait over a longer time period (13, 15, and 20 weeks), so the birds could be at different growing periods compared to our study (25). Many parameters also had age and main effects interactions including total double support time, stride length, step velocity, step time, stance time, gait cycle time, and impulse. In this study, an age and treatment foot interaction was observed for step length, and maximum force. This suggests that treatment may have more or less of an effect depending on the age of the bird. For some gait parameters, even the foot the treatment was applied to can affect gait.

The validity of accelerometers is determined by both accuracy and specificity, while reliability refers to the precision and sensitivity (37, 38). Validity reflects how well the accelerometers measure the true step counts. In contrast, the reliability of the accelerometers reflect how much error is present when determining step counts. Compared to previous studies utilizing accelerometers, the AXY-3 Data Loggers used in this study were comparable in terms of accuracy, but had a higher false discovery rate (8, 17, 33, 39). The false discovery rate increased as birds aged with the highest values reported at 16 weeks. These high false discovery rates could be due to the increased variation among the individual birds as they aged or alternatively, the processing method used to smooth the data in LabVIEW. One method to reduce the false discovery rate would be to adjust the step threshold to each individual turkey rather than using an average step threshold for all birds of a certain age. However, this would not be feasible for large numbers of birds. The validity and reliability of these accelerometers was also greatly affected by non-habituated birds shaking and pecking at the accelerometers on their legs during data collection. When examining data from habituated and non-habituated turkeys, 35 out of the 44 false positives were attributed to the non-habituated birds, further demonstrating the importance of habituation when using accelerometers to detect the stepping activity of turkeys. The morphology of birds might also have contributed to the higher false discovery rate at 16 weeks.

Accelerometers have been shown to provide a use for both scientific studies and commercial uses in animals. By showing the potential validity of AXY-3 Data Loggers, steps can be taken to study how stepping behavior and activity level changes can be indicators of welfare issues such as lameness.

Although the sample size was small, which had the potential to greatly affect the results of this study, results are still helpful in contributing to the lack of research regarding uses of micro accelerometers for poultry. Balancing the treatment between left and right foot also provided an extra factor to consider in the already low power of the results. Further research should be conducted on the long term uses of micro-accelerometers to detect behaviors and welfare concerns.

## Conclusion

Based on the results, AXY-3 Micro Accelerometers are an effective tool for recording the stepping activity of turkeys, but the reliability and validity of these accelerometers varied by bird age and prior habituation to the bandages used to secure the accelerometers. Most gait parameters in turkeys are sensitive to age effects, and unhabituated birds were shown to have an additional age treatment interaction further affecting gait. Turkey health status and body condition scores were affected by age and not by treatment group, with feather condition worsening as birds aged. Based on our results, a one-week habituation period using only a bandage is effective in habituating turkeys to wearing micro-data loggers. Further steps should be taken to assess the uses of activity level related behaviors in turkeys to determine if stepping behavior can be used as a proxy for changes in behavior. Furthermore, future studies should look into age-related gait dynamics and male and female gait changes.

## Data Availability Statement

The raw data collected for this study are available upon request to qualified research scientists.

## Author Contributions

ME designed the project, assisted in data collection and analyses, and made edits to the manuscript. HD assisted in methods, analysis of accelerometer output and provided edits to the manuscript. RS collected and analyzed data and drafted the manuscript.

### Conflict of Interest Statement

The authors declare that the research was conducted in the absence of any commercial or financial relationships that could be construed as a potential conflict of interest.
